# In vitro cell culture of patient derived malignant pleural and peritoneal effusions for personalised drug screening

**DOI:** 10.1186/s12967-020-02331-x

**Published:** 2020-04-10

**Authors:** Cheng-Guang Wu, Francesca Chiovaro, Alessandra Curioni-Fontecedro, Ruben Casanova, Alex Soltermann

**Affiliations:** 1 Institute of Pathology and Molecular Pathology, University Hospital Zurich, Schmelzbergstrasse 12, 8091 Zurich, Switzerland; 2 University of Zurich, Rämistrasse 71, 8006 Zurich, Switzerland; 3 InSphero AG, Wagistrasse 27, 8952 Schlieren, Switzerland; 4 Department of Medical Oncology and Haematology, University Hospital Zurich, Rämistrasse 100, 8091 Zurich, Switzerland; 5 ADMED Pathology, Rue de la Maladière 45, 2000 Neuchâtel, Switzerland

**Keywords:** Malignant effusion, Cytology, Cell culture, Immunotherapy, Drug screening, PD-L1

## Abstract

**Background:**

Malignant serous effusion (MSE) denotes a manifestation of metastatic disease with typical high concentrations of both cancer and immune cells, making them an ideal resource for in vitro cytologic studies. Hence, the aim of the study was to investigate the features of 2D and 3D MSE culture systems as well as their feasibilities for in vitro drug screening.

**Methods:**

Pleural and peritoneal effusions from 8 patients were collected and processed for 2D monolayer and 3D hanging drop cell culture into GravityPLUS™ plates. Representative markers for cell components, proliferation rate and tumour classification were investigated by immunohistochemistry, followed by absolute quantification using a digitalised image analysis approach. Further, we implemented another 3D cell culture model based on a low attachment method for in vitro drug sensitivity testing of carboplatin, pemetrexed and pembrolizumab for 5 patients.

**Results:**

Monolayer cell culture was favourable for the growth of mesothelial cells, while hanging drop culture in GravityPLUS™ plates showed better ability for preserving cancer cells, inducing positive diagnostic markers expression and restraining the growth of mesothelial cells. For in vitro drug testing, MSE from five patients presented various drug sensitivities, and one case showed strong response to PD-1 checkpoint inhibition (pembrolizumab). For some patients, the application of combinatorial drugs had better therapeutic responses compared to monotherapy.

**Conclusions:**

Digitalised quantification of data offers a better understanding of different MSE culture models. More importantly, the proposed platforms are practical and amenable for performing in vitro chemo-/immunotherapeutic drug testing by using routine cytologic MSE in a personalised manner. Next to cell blocks, our work demonstrates the prognostic and predictive value of cytologic effusion samples.

## Background

Heart failure, infection and malignancy are the main causes of serous effusions [1]. The conventional smear (CS) and cell block (CB) methods are commonly used for cytologic diagnosis of exudative serous effusions [2], and cytopathologists frequently use these tools for gaining immediate information regarding malignancy or specific infection in patients in which organ biopsies are difficult to perform. Patients bearing malignant serous (pleural and peritoneal) effusions have diverse median overall survivals between 4 and 9 months depending on cancer entity [3–5]. Etiologically, adenocarcinomas of lung, breast, ovary as well as mesothelioma are the most frequent cancer entities that metastasise to the pleural and peritoneal cavity [5, 6].

In the MSE, cancer-associated cells such as activated mesothelial cells and immune cells could promote and support cancer cell survival and proliferation without the need of stromal cells. Thanks to the abundant access to ligands and growth factors, malignant cells in MSE can spread and metastasise to adjacent sites [7]. These cells are usually creating a pro-inflammatory environment enriched in cytokines and growth factors promoting a permissive local microenvironment for metastatic processes. With the prominent intratumoural heterogeneity, MSE is capable of driving disease progression towards an invasive phenotype [8]. We have previously shown that patients bearing malignant pleural effusions were characterised by heterogeneous expression of immune cells and immunomodulators in their effusion liquids, which resulted in different prognosis [9].

Chemotherapy by intracavitary administrating of anticancer drugs is a common method for treating MSE [10–12]. However, only modest gains have been made in long-term patient survival due to multidrug-resistant and highly aggressive characteristics of tumours growing in either pleural or peritoneal cavity. Screening of patients’ own samples for in vitro chemo/immunotherapeutic agent selection enables the optimisation of individual therapeutic regimens [13]. To date, the utilisation of MSE 2D cell culture for drug screening and other experimental studies has already been demonstrated from literature evidences [8, 13, 14]. Moreover, in vitro MSE models generated either with scaffold-free techniques or with hydrogel matrix support were also used in few studies [15, 16]. There are still some efforts that need to be done, such as to better retain tumour heterogeneity and cellular components, and to establish a more robust and standardised platform for preclinical testing.

Therefore, the aim of our study was to perform a cytologic analysis using a digital image analysis approach to investigate the alterations of diagnostic marker profiles, cell components and cell proliferation rates of MSE in 2D and 3D cell cultures. Ultimately, we wanted to evaluate the feasibility of MSE models for personalised drug test.

## Methods

### Collection and processing of cells

Only MSE with unequivocal diagnostic results from the department of pathology were accepted including the tumour types of lung adenocarcinoma (LADC), breast carcinoma (Breast-Ca), ovarian carcinoma (Ovarian-Ca), gastro-intestinal carcinoma (GIT-Ca) and melanoma. After centrifugation the supernatant was removed and preserved. The cellular sediment was resuspended with the RPMI 1640 culture medium (Gibco, Waltham, MA, USA) followed by a cell-counting step using NucleoCounter (ChemoMetec, Denmark).

For 2D cell culture, 1 million cells were cultured in a T-25 culture flask (Corning, NY, USA) in an incubator at 37 °C and 5% CO_2_ with 10 ml complete culture medium RPMI 1640 with 10% fetal calf serum (FCS, Gibco) and Antibiotic–Antimycotic^®^ (Gibco). The medium was replaced every 3–5 days. After reaching 70–80% confluence, the cells were harvested by incubation with TrypLE Express (Gibco) for up to 5 min at 37 °C.

For 3D hanging drop culture, GravityPLUS™ plates (InSphero, Schlieren, Switzerland) were used as described previously [17]. Briefly, MSE cells were seeded at a density of 1000 cells per drop and co-cultured with normal human dermal fibroblasts (nHDF) at a ratio of 1:1 in an InSphero Proprietary medium at 37 °C and 5% CO_2_. After 2–4 days of spheroid formation, microtissues were transferred into GravityTrap™ ULA plates (InSphero) for further analysis.

For the low-attached 3D cell culture, the cell pellet was resuspended in 50% of complete culture medium and 50% MSE supernatant and seeded in a cell culture dish (100 mm). Since MSE samples were normally preserved at 4 °C before processing, we put the dish at 37 °C and 5% CO_2_ in the incubator for 24 h on a rotator (30 rpm) to mimic the motion as the MSE cells would experience in the patient’s cavity. Afterwards, the cells were dispensed into a 96 well plate with ultra-low attachment surface (Corning) at a density of 7000 cells per well in complete culture medium overnight, for further in vitro drug screening assays.

### Cell blocks and immunohistochemistry (IHC)

Cell pellets from harvested 2D cultured cells and original MSE effusions were prepared and processed to make cell blocks as previously described [18]. Briefly, cell pellets were supplemented with thrombin and plasma for clot formation. After 4% para-formaldehyde fixation for 1 h at 4 °C, clots were paraffin-embedded and haematoxylin-eosin (H&E) stained. Microtissues were collected in 1.5 ml tubes and washed once with PBS. Consequently, they were fixed in 4% para-formaldehyde for 1 h at 4 °C. Fixed microtissues were collected in the tip of a 1.5 ml microtube, embedded in 2% agarose (Amresco, Solon, OH) and covered with PBS. For paraffin-embedding, the agarose plugs were taken out of the microtubes and the tip containing the microtissues was cut and placed in formalin for 12–14 h, followed by gradual dehydration. Finally, the plugs were embedded in paraffin (microtissues facing downwards) in order to facilitate sectioning.

For IHC analysis, Sects. (3 μm) were prepared and stained for antibodies against CD3 (T cells), CD45 (immune cells), Pan-CK (epithelial tumour cells), calretinin (mesothelial cells), MIB-1 (cell proliferation marker), and diagnostic markers for each MSE tumour entity: thyroid transcription factor 1 (TTF-1, LADC), CDX2 (GIT-Ca), estrogen receptor (ER, Breast-Ca and Ovarian-Ca), melan-A and S100 (melanoma) were performed on a Benchmark Ultra platform (Roche, Ventana Medical Systems, Oro Valley, AZ, USA) with protocols used for routine diagnostics. PD-L1 antibody clone E1L3N (Cell Signaling Technology, Danvers, MA, USA) was used. PD-L1 immunoreactivity was dichotomised into low (0 to 49%) and high (≥ 50%), taking into account only membranous staining of tumour cells. Diagnostic markers were scored 0 (negative −) or 1 (positive +). All primary antibodies used for IHC analysis were listed in Additional file 2: Table S1.

### Digital image analysis

Immunohistochemically stained sections were scanned with a high-resolution whole-slide scanner (Hamamatsu Nanozoomer Digital Pathology) using a ×40 objective with spatial resolution of 0.23 µm/pixel. As for the colour-based segmentation, IHC results were quantified using ImageJ software (National Institutes of Health, USA, version 1.47t), which could separate positive stained areas (brown/red) from non-stained areas (blue/grey) by colour thresholding using the Lab colour space. Fixed thresholds were used for each set of images. The ratio of positive pixels to the total number of pixels per image was quantified (n = 3).

### In vitro drug test

Drug efficacy test was applied only to the low attached cell culture systems. Drug sensitivities of carboplatin (Sigma-Aldrich, St. Louis, MO, USA), pemetrexed (Santa Cruz Biotechnology, Dallas, TX, USA) and pembrolizumab (KEYTRUDA^®^, Merck & Co., USA) were evaluated. There were six testing groups including (1) carboplatin (50 µM), (2) pemetrexed (5 µM), (3) pembrolizumab (2.5 nM), (4) carboplatin (50 µM) + pemetrexed (5 µM), (5) carboplatin (50 µM) + pemetrexed (5 µM) + pembrolizumab (2.5 nM) and (6) control group (complete culture medium only), (n = 4). After incubation for 48 h, cell proliferation was assessed using CCK-8 kit (Dojindo, Japan). Briefly, 10 µl of CCK-8 was added into each testing well (containing 100 µl medium), followed by incubation for one hour at 37 °C and 5% CO_2_. The optical density was determined using a spectrophotometer (Infinite F Plex, Tecan, Switzerland) at 490 nm with background correction at 630 nm.

### Statistical analysis

All data were expressed as mean ± SD. All statistical analyses were performed on SPSS software, version 23 (IBM, USA) or environment R, version 3.4.2 (R Core Team). Results were analysed with Student’s *T* test and p-values < 0.05 were considered statistically significant.

## Results

### Cohort description

We received 17 malignant effusions from pleura or ascites, among which 13 samples (Table 1) fulfilled the standards for proper cell cultures. Samples from patients 1 to 8 (P1 to P8) were used for 2D and 3D hanging drop cell cultures, and subsequently analysed for the expression of markers to discriminate the different cell components. Samples P9 to P13 were processed only for in vitro drug testing. Clinical information together with sample volume and cell concentration for each effusion are listed in Table 1.

**Table 1:** Cohort description

Case	Location	Age	Sex	Tumour type	Diagnostic markers	Volume (ml)	Cell count (M/ml)	Drug test
1	Pleura	60	Male	GIT-Ca	CDX2+	45	2.3	No
2	Pleura	56	Male	Melanoma	Melan-A+	2600	2.5	No
3	Pleura	71	Female	GIT-Ca	CDX2+	2000	4.2	No
4	Ascites	67	Male	GIT-Ca	CDX2+	1800	0.6	No
5	Ascites	46	Female	Breast-Ca	ER+	600	6	No
6	Ascites	68	Female	Melanoma	Melan-A−, S100+	2000	1.4	No
7	Pleura	43	Male	GIT-Ca	CDX2+	1200	0.38	No
8	Pleura	92	Male	LADC	TTF-1+	800	0.38	No
9	Pleura	54	Female	LADC	TTF-1+	1600	0.03	Yes
10	Ascites	68	Female	Ovarian-Ca	ER+	2500	0.08	Yes
11	Pleura	43	Female	Ovarian-Ca	ER+	1100	0.12	Yes
12	Pleura	72	Male	LADC	TTF1−	1500	0.5	Yes
13	Pleura	65	Female	Breast-Ca	ER+	2000	0.4	Yes

*TTF*-*1* thyroid transcription factor 1, *ER* estrogen receptor, *LADC* lung adenocarcinoma, *Breast*-*Ca* breast carcinoma, *Ovarian*-*Ca* ovarian carcinoma, *GIT*-*Ca* gastrointestinal carcinoma, + positive, − negative

### Establishing MSE in vitro cell culture systems

**Fig. 1:**
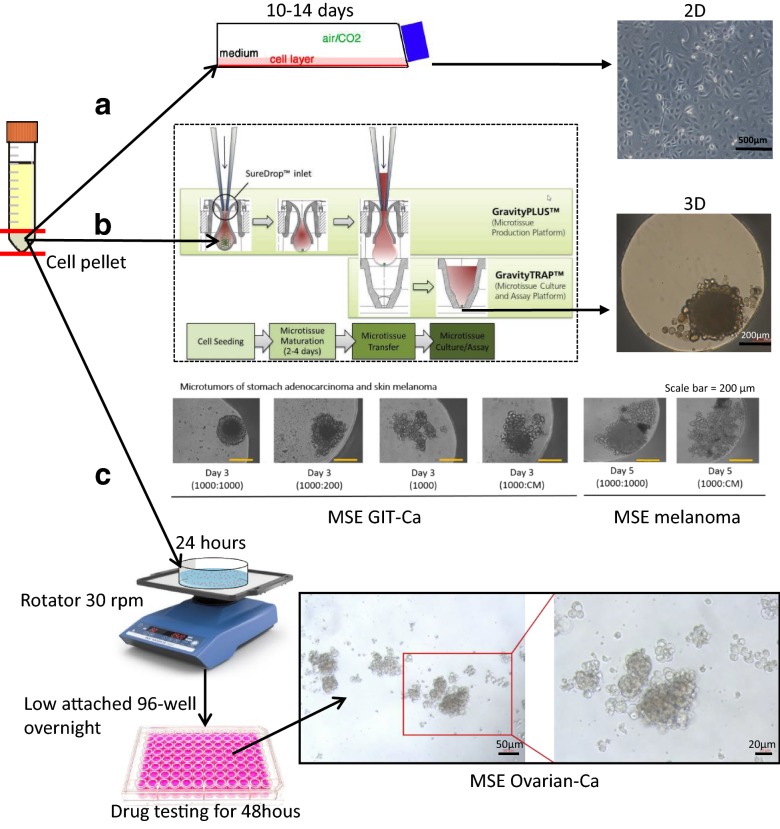
Malignant serous effusions (MSE) 2D and 3D cell culture and drug screening system. **a** conventional 2D monolayer cell culture system, scale bar = 500 µm. **b** 3D hanging drop cell culture system (modified from InSphero Product Manual of GravityPLUS™ Hanging Drop System with the permission from InSphero AG). For the two show cases of MSE stomach adenocarcinoma and skin melanoma, the ratio indicated the number of MSE cells to normal human dermal fibroblasts (nHDF), scale bar = 200 µm. **c** drug screening platform using low attached cell culture system, scale bar = 50 µm (left) or 20 µm (right), respectively. *CM* conditional medium

We investigated one conventional 2D cell culture and two 3D cell culture systems. After 2D cell culture of MSE over 10 to 14 days, cells reached 70–80% confluence (Fig. 1a). 3D hanging drop cell culture system offered an efficient approach for microtumour formation (diameter around 200 µm) with the help of nHDF to provide physical support and enhance cellular spheroids assembly. Several ratios of MSE cells and nHDF have been tested with the final indication of 1:1 as the optimal one. MSE cells could not form compact microtissue without the support of nHDF (Fig. 1b). In order to reproduce the microenvironment of MSE in vivo conditions, we created a platform containing a rotation process, following floating cell cultures using ultra low attachment wells (Fig. 1c). Considering the short half-life of immune cells and the time needed for potential clinical applications, the drug test was only performed for 48 h. There were cell clots floating in the medium, which were surrounded by several single cells.

### H&E, immunohistochemistry and digital image analysis

**Fig. 2:**
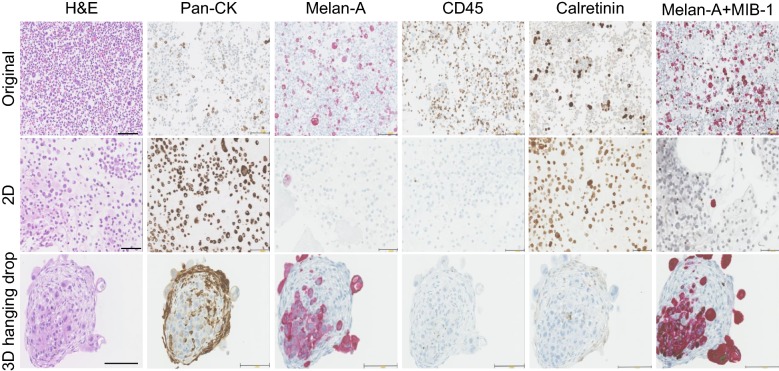
Comparison of H&E and expression of representative markers for cell types, diagnosis and proliferation. Original melanoma MSE and the respective 2D and 3D cell cultures were compared. Pan-CK (tumour cells), CD45 (immune cells) and calretinin (mesothelial cells) positive expression can be visualised in brown stain. Double staining of Melan-A (melanoma diagnostic marker) and MIB-1 (proliferation marker) are detected, respectively, in red and brown stains, scale bar = 100 µm

**Fig. 3:**
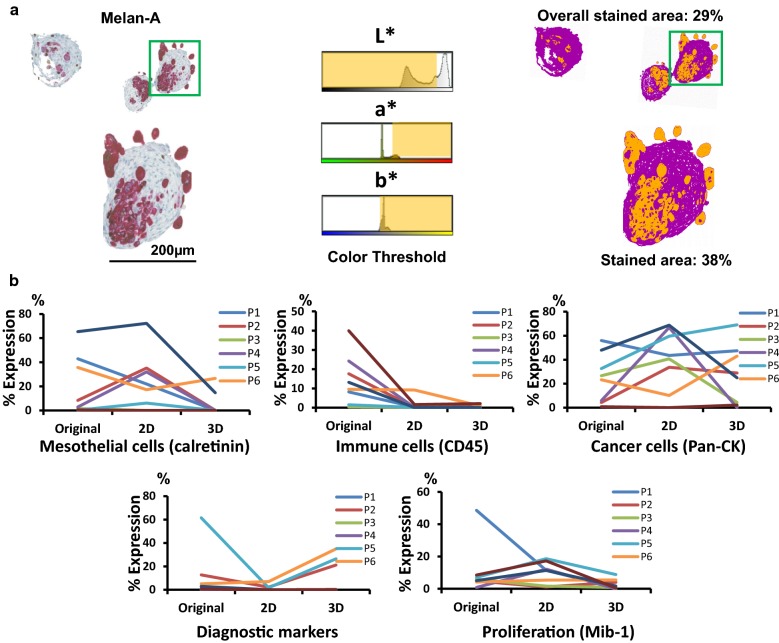
Quantification of IHC staining. **a** Digitalised image analysis approach of IHC staining, a showcase of Melan-A IHC. Melan-A staining of the showcase (P2) sample in 3D hanging drop cell culture. The positive staining area (red) recognised by colour thresholding using the “Lab” colour space was annotated in yellow; non-staining area (blue/grey) was annotated in purple, scale bar = 200 µm. **b** Quantification of expressed histological markers for proliferation, diagnosis and representative cellular components in original MSE, 2D and 3D hanging drop cell cultures. The ratio (percentage) of positive pixels to the total number of pixels of each marker was presented

The histological analysis based on cell types, cell proliferation and diagnostic markers are shown in Fig. 2 (showcase of P2, melanoma). The IHC yielded good immunoreactivity performance with clear background for all markers investigated. However, 2D and 3D culture systems had a different impact on the expression of IHC markers and the overall cell growth rate. In order to quantify these marker expressions, we performed digitalised image analysis (showcase P2, Fig. 3a). The positive staining area (red) recognised by colour thresholding using the “Lab” colour space was annotated in yellow; non-staining area (blue/grey) was annotated in purple. Other IHC stainings with positive area in brown were processed similarly (Additional file 1: Fig. S1).

### Comparison of cellular components, cell proliferation and diagnostic markers expression between 2D and 3D cell culture systems

Histological analysis of cell types, cell proliferation and diagnostic marker expression were quantified and presented as the ratio of positive pixels to the total number of pixels per image (Fig. 3b, Additional file 2: Table S2). Overall, 2D culture condition sustained the growth of mesothelial cells (calretinin) and poorly supported the retainment of diagnostic markers (P2, P4 and P7). By contrast, 3D hanging drop culture showed better preservation of cancer cells with positive diagnostic marker expression (P2, P5 and P6). However, both culture systems were not suitable for the growth of immune cells, with poor-negative staining for CD45. Additionally, 2D culture condition was more favourable to the general cell growth.

### In vitro drug sensitivity test of MSE with chemotherapeutic agents and PD-1 blockade with immunomodulatory drug

**Fig. 4:**
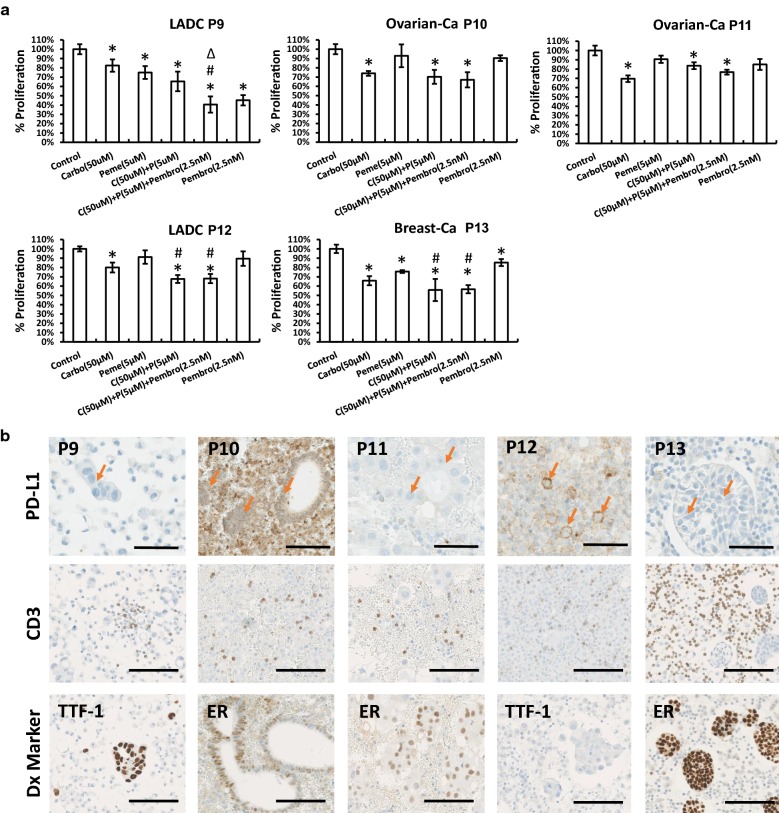
In vitro drug sensitivity test using low attached 3D cell culture system. **a** MSE samples were treated with carboplatin (50 µM), pemetrexed (5 µM) and pembrolizumab (2.5 nM) in a single or combinatorial manner, N = 4. Cell proliferation readout of each group was shown as value ± SD compared with the corresponding vehicle group (control group). *p-value < 0.05 other groups compared with the control group; ^#^p-value < 0.05 combinatorial test groups compared with Carbo and Peme test groups; ^∆^p-value < 0.05 C + P + Pembro triple test group compared with C + P combinatorial test group. **b** PD-L1, CD3 and diagnostic (Dx) marker staining of original cell blocks, scale bar = 100 µm. Arrow: tumour cell. Abbreviations: carboplatin (carbo, C), pemetrexed (peme, P), pembrolizumab (pembro)

As shown in Fig. 4a, patients responded heterogeneously to traditional chemotherapeutic agents at the testing concentration levels. Especially, upon administration of carboplatin and pemetrexed, cases P9 and P13 were the most affected with high level of cytotoxicity. In contrast, P10, P11 and P12 were more sensitive only to carboplatin, showing a strong impairment in cell proliferation, whereas pemetrexed had only minor effect to the cells. Notably, combinatorial treatment with carboplatin and pemetrexed displayed higher cytotoxic effect in P12 and P13 rather than in single drug treatment regimens.

For the applications of immunotherapy treatment based on pembrolizumab, P9 was selected as the best case, showing a positive therapeutic response with a remaining 40% of cell viability, while other patients (P13 was slightly significant) did not show significant sensitivity towards the biological treatment. Moreover, all five malignant effusions were highly infiltrated by CD3^+^ T cells (original cell blocks, Fig. 4b, the quantification result in Additional file 2: Table S3). In addition, PD-L1 was heterogeneously expressed, with high level in P12 and P10 (non-membranous positive staining) and low level in P9, P11 and P13 (Fig. 4b). Furthermore, a triple combination of small molecules (carboplatin and pemetrexed) with biologic anti-PD-1 pembrolizumab considerably enhanced the cytotoxicity in P9 compared to treatment with only small molecules. This specific outcome was not observed in any of the other samples.

## Discussion

We propose our in vitro drug screening platform from MSE material as reliable alternative to invasive in vivo research with increased cost effectiveness. The MSE system enables the assessment of several chemotherapeutic and biological drugs already approved and it can be considered as immediate biological tool for exploring novel therapeutic approaches.

However, the current preclinical models are not sufficiently developed to account for the intratumoural heterogeneity and inter-patient variability [19]. Therefore, we first investigated 2D and 3D hanging drop cell culture systems using MSE samples from several cancer entities and studied their features. According to the literatures, monolayer cell cultures show some weakness related to the overgrowth of fibroblast (solid tumour), long culturing time and loss of cancer cell heterogeneity [19, 20]. In our study, despite the high growth rate and high component of Pan-CK positive cells of 2D cell cultures, most of the cells lost the tumour phenotype. Research from Ordóñez N.G. shows that nearly all mesothelial cells (including mesotheliomas) can be positive for pan-cytokeratin (Pan-CK) (including AE1/AE3) [21]. Therefore, for MSE cytologic diagnosis, Pan-CK is not be a proper marker for recognising tumour cells. Clearing of mesothelial cells using magnetic cell separation (MACS systems) might be crucial to establish pure cultures of MSE to be used for screening assays [13].

The optimal growth conditions observed in 3D cell cultures supported the proliferation of tumour cells with a more reduced presence of mesothelial cells. The proportion of cells expressing diagnostic markers was also higher than corresponding 2D culture mirroring the histological analysis of the original MSE samples. The use of 3D Gravity plates enables high throughput chemo drug screening, and it allows specific functional studies involved in cancer progression. Despite these advantages, MSE as a liquid biopsy will require a fibroblast component for an easier cell aggregation in a 3D structure. As both 2D and 3D hanging drop systems lost immune cell viability for the necessary screenings, we thereafter established the drug testing platform with a processing time less than 4 days. It mirrored the floating cells culture property of MSE microenvironment, which might better preserve the viability of immune cells and augment the chance for tumour–immune cells interaction.

Carboplatin and pemetrexed were selected for the therapeutic drug-testing assay, as they are frequently used in the treatment of lung adenocarcinoma, breast cancer and mesothelioma. In addition, we tested the anti-PD-1 drug pembrolizumab, which has been widely used for many metastatic cancer therapies [22, 23]. Drug concentrations were selected and designed according to available scientific information (125514Orig1s000, FDA pembrolizumab in vitro IC50 test) [24, 25]. Different grades of sensitivity were observed in all patients following single or combinatorial drug treatments. Interesting, most of our in vitro outcome could be positively correlated with the benefits of current therapeutic approaches based on the simultaneously targeting of different signalling pathways and empowering the immune system using different immunomodulators [26–28]. In our investigation, we could not observe any upregulation of PD-L1, which is refereed as a predictive marker to screen patients for the applications of immunotherapy.

Further open questions are to be addressed concerning the pembrolizumab efficacy and function in this system, given the fact that a direct co-culture with patient’s autologous lymphocytes is difficult to achieve, and the optimisation of digitalised image analysis, which was conducted based on pixels but not cell counts in our study.

## Conclusion

Our first data encourage and prompt us towards further investigation of MSE to uncover complex mechanisms of drug action, while the system provide a good platform for selection of drug candidates. Culture of MSE in 3D structure better reflects the original cell composition and functions, therefore offering a better clinical translation of in vitro observations.

## Supplementary information

**Additional file 1: Fig. S1.** Digital image analysis of IHC staining of S100 and ER. (A) S100 staining of the showcase (P6) sample after 3D hanging drop culture. (B) ER staining of the showcase (P5) sample after 3D hanging drop culture. Positive staining area (brown) was recognised by colour thresholding using the “Lab” colour space and was annotated in yellow; non-staining area (blue/grey) was annotated into purple, scale bar = 200 µm.

**Additional file 2: Table S1.** List of antibodies used for immunohistochemistry (IHC) studies. Abbreviations: thyroid transcription factor 1 (TTF-1), estrogen receptor (ER). **Table S2.** Quantification results of markers for proliferation, diagnosis and representative cellular components. The ratio of positive pixels to the total number of pixels of each marker measured by digitalised image approach was presented as the percentage expression ± SD (n = 3). **Table S3.** Quantification result of CD3+ immune cells.

## Data Availability

All data generated or analysed during this study were included in this published article and its additional files.

## Abbreviations

MSE: Malignant serous effusion
CS: Conventional smear
CB: Cell block
LADC: Lung adenocarcinoma
Breast-Ca: Breast carcinoma
Ovarian-Ca: Ovarian carcinoma
GIT-Ca: Gastro-intestinal carcinoma
IHC: Immunohistochemistry
FCS: Fetal calf serum
nHDF: Normal human dermal fibroblasts
H&E: Haematoxylin–eosin
TTF-1: Thyroid transcription factor 1
ER: Estrogen receptor
CM: Conditional medium
Pan-CK: Pan-cytokeratin
