# Supplementary figures and images for: In vitro cell culture of patient derived malignant pleural and peritoneal effusions for personalised drug screening
Source: J Transl Med. 2020 Apr 10;18:163. doi: 10.1186/s12967-020-02331-x (PMC7149866; doi:10.1186/s12967-020-02331-x)

Figure S1

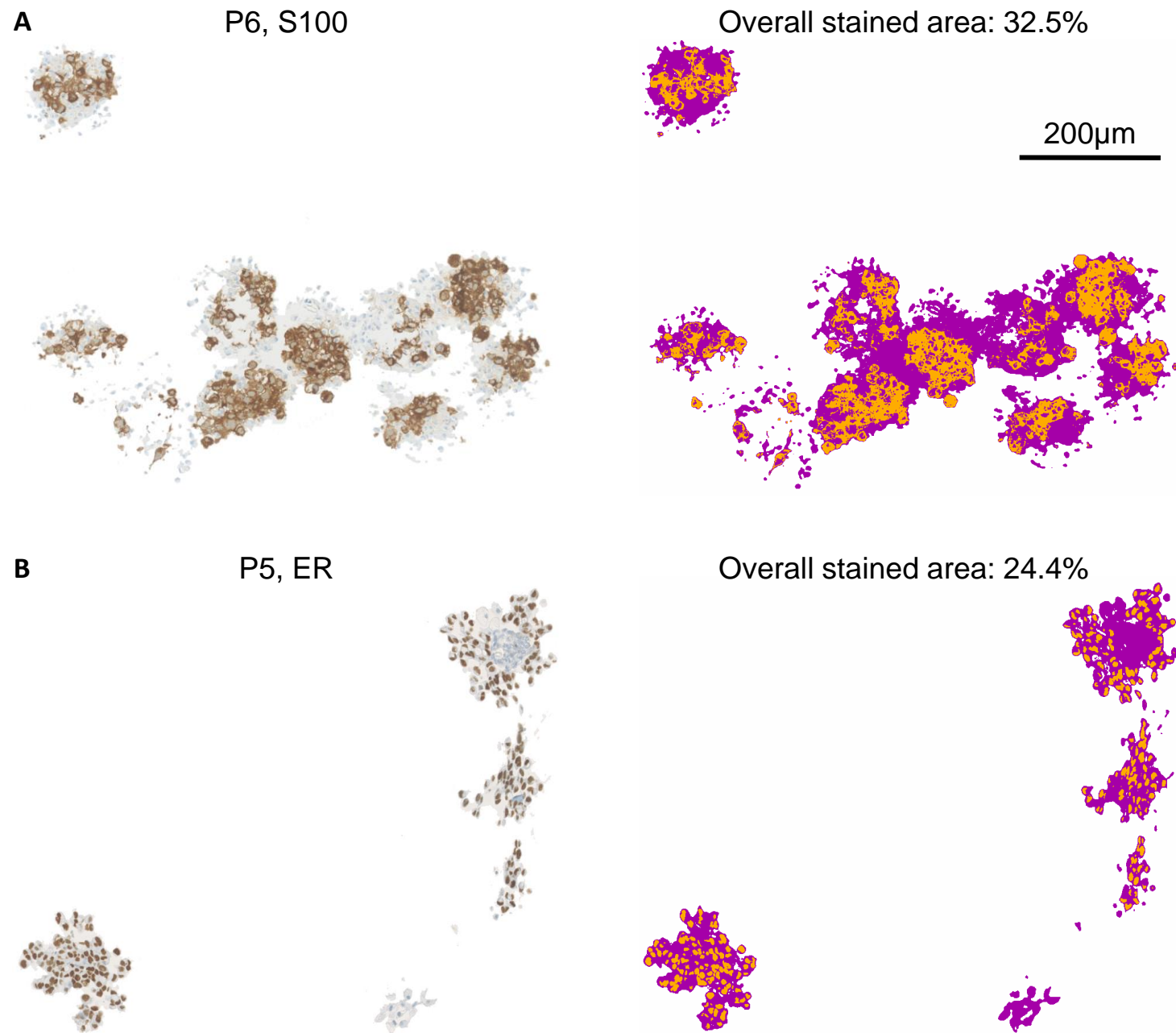

Supplement: Supplementary file 1 — Additional file 1: Fig. S1. Digital image analysis of IHC staining of S100 and ER. (A) S100 staining of the showcase (P6) sample after 3D hanging drop culture. (B) ER staining of the showcase (P5) sample after 3D hanging drop culture. Positive staining area (brown) was recognised by colour thresholding using the “Lab” colour space and was annotated in yellow; non-staining area (blue/grey) was annotated into purple, scale bar = 200 µm. [file 12967_2020_2331_MOESM1_ESM.pdf]
